# *Brucella* Manipulates Host Cell Ferroptosis to Facilitate Its Intracellular Replication and Egress in RAW264.7 Macrophages

**DOI:** 10.3390/antiox13050577

**Published:** 2024-05-08

**Authors:** Guangdong Zhang, Hai Hu, Yi Yin, Mingxing Tian, Zhigao Bu, Chan Ding, Shengqing Yu

**Affiliations:** 1 Shanghai Veterinary Research Institute, Chinese Academy of Agricultural Sciences (CAAS), Shanghai 200241, China; gdzhang688@foxmail.com (G.Z.); wuhusihaixh@163.com (H.H.); yinyisonia@126.com (Y.Y.); tianmx530@126.com (M.T.); 2 Harbin Veterinary Research Institute, Chinese Academy of Agricultural Sciences (CAAS), Harbin 150069, China; buzhigao@caas.cn; 3 Jiangsu Co-Innovation Center for Prevention and Control of Important Animal Infectious Diseases and Zoonoses, Yangzhou 225009, China

**Keywords:** *Brucella*, ferroptosis, glutathione peroxidase 4 (GPX4), GTP cyclohydrolase1 (GCH1), intracellular replication, bacterial egress

## Abstract

*Brucella* virulence relies on its successful intracellular life cycle. Modulating host cell death is a strategy for *Brucella* to survive and replicate intracellularly. Ferroptosis is a novel regulated cell death characterized by iron-triggered excessive lipid peroxidation, which has been proven to be associated with pathogenic bacteria infection. Thus, we attempted to explore if smooth-type *Brucella* infection triggers host cell ferroptosis and what role it plays in *Brucella* infection. We assessed the effects of *Brucella* infection on the lactate dehydrogenase release and lipid peroxidation levels of RAW264.7 macrophages; subsequently, we determined the effect of *Brucella* infection on the expressions of ferroptosis defense pathways. Furthermore, we determined the role of host cell ferroptosis in the intracellular replication and egress of *Brucella*. The results demonstrated that *Brucella* M5 could induce ferroptosis of macrophages by inhibiting the GPX4-GSH axis at the late stage of infection but mitigated ferroptosis by up-regulating the GCH1-BH4 axis at the early infection stage. Moreover, elevating host cell ferroptosis decreased *Brucella* intracellular survival and suppressing host cell ferroptosis increased *Brucella* intracellular replication and egress. Collectively, *Brucella* may manipulate host cell ferroptosis to facilitate its intracellular replication and egress, extending our knowledge about the underlying mechanism of how *Brucella* completes its intracellular life cycle.

## 1. Introduction

Brucellosis is a worldwide-distributed zoonosis that severely threatens human health and animal husbandry [1,2,3,4]. It is estimated that there are approximately 1.6 to 2.1 million cases of human brucellosis each year but the true incidence may be several times higher [5,6,7]. Brucellosis remains a leading neglected zoonotic disease globally, especially in the developing world [8].

*Brucella* spp., the etiological agents of brucellosis, are Gram-negative facultative intracellular bacteria, which can infect a range of cell types, such as monocytes, macrophages, dendritic cells, and trophoblast cells [9]. Evidence supports that the virulence of *Brucella* is completely dependent on its ability to survive and replicate in host cells [9,10,11]. As a stealthy pathogen, *Brucella* has evolved numerous strategies to defend host bactericidal responses and then establish intracellular multiplication niches [12,13]. It has been reported that *Brucella* infection induces the premature death of neutrophils to promote their phagocytosis by macrophages [14,15]. Nevertheless, *Brucella* infection prevents the cell death of monocytes, lymphocytes, macrophages, and lung epithelia cells as infected cells show resistance to spontaneous and induced cell death, which is contrary to non-infected cells [16,17,18,19,20,21]. Strikingly, the non-infected cells adjacent to the *Brucella*-infected cells also showed resistance to spontaneous and induced cell death [16]. These findings suggest that modulating host cells’ death may be an important strategy used by *Brucella* to benefit its infection [17].

Ferroptosis is a specific form of regulated cell death triggered by iron-dependent aberrant lipid peroxidation [22,23,24]. Dysregulated iron homeostasis triggers the accumulation of labile iron pool, the intracellular nonprotein-bound redox-active iron, which feeds iron-catalyzed reactive oxygen species (ROS) production [23]. Through the Fenton reaction, ferrous iron is oxidized to ferric iron while catalyzing the formation of hydroxyl radicals, which are particularly preferred to peroxide polyunsaturated fatty acids (PUFAs) [25,26]. PUFA-containing phospholipids are the main components of the cell membrane; therefore, the cell membrane is easily peroxided by iron-catalyzed ROS, which leads to the accumulation of toxic reactive aldehydes, such as malondialdehyde (MDA) and 4-Hydroxynonenal (4-HNE), and subsequent causes membrane rupture [23,27]. Hence, the cellular surveillance and defense systems for ferroptosis are essential for host cell homeostasis [24]. Up to now, a few of these systems have been identified. Glutathione peroxidase 4 (GPX4) is a selenocysteine-containing oxidoreductase and is the sole oxidoreductase that directly reduces phospholipid hydroperoxides (PLOOH), the executor of lipid peroxidation, to non-toxic lipid alcohols with the expense of glutathione (GSH) [26,28,29]. The biosynthesis of GSH was dependent on the cystine imported from the extracellular environment by a transmembrane heterodimer consisting of solute carrier family 7 member 11 (SLC7A11) and solute carrier family 3 member 2 (SLC3A2) [22]. Given its indispensable activity in defending against lipid hydroperoxides, the GPX4-GSH pathway is recognized as the most central one of the ferroptosis regulation systems [24,29]. Ferroptosis suppressor protein 1 (FSP1), previously known as apoptosis-inducing factor mitochondrial 2 (AIFM2), is a GPX4-independent ferroptosis suppressor. Research reported that FSP1 can block gpx4 depletion-induced ferroptosis by reducing coenzyme Q10 (CoQ10) to CoQH2, a radical-trapping antioxidant (RTA) that inhibits membrane lipid peroxidation through detoxifying lipid peroxyl radicals [30,31]. Mao et al. identified a mitochondrial dihydroorotate dehydrogenase (DHODH)-mediated ferroptosis defense system parallel to the SLC7A11-GPX4-GSH axis [32]. In the mitochondrial inner membrane, DHODH reduces CoQ10 to CoQH2 to suppress lipid peroxidation. Using a genomic CRISPR screening method, GTP cyclohydrolase1 (GCH1) was identified as another GPX4-independent defender against ferroptosis [33,34]. GCH1 is the rate-limiting enzyme of the biosynthesis of tetrahydrobiopterin (BH4), which is an RTA that functions analogously to CoQ10 to scavenge lipid peroxyl radicals [35].

More and more evidence suggests a broad relationship between ferroptosis with diseases, including bacterial infectious diseases [36,37,38]. For instance, *Mycobacterium tuberculosis* (*M. tuberculosis*) infection induces host ferroptosis to facilitate its dissemination [39,40]; *Pseudomonas aeruginosa* (*P. aeruginosa*), *Escherichia coli* (*E. coli*), *Salmonella enteritidis* (*S. enteritidis*), and *Staphylococcus aureus* (*S. aureus*) infections trigger host ferroptotic stress [41,42]. Our previous study showed that a *Brucella* rough mutant strain induced macrophage ferroptosis at the early stage of infection [43]. However, the mechanism underlying the *Brucella*–host ferroptosis interaction and the role of host ferroptosis in *Brucella* infection remain unknown. The rough-type *Brucella* strains defect in intracellular replication and most of the virulent *Brucella* strains, such as *Brucella abortus* (*B. abortus*), *Brucella suis* (*B. suis*), and *Brucella melitensis* (*B. melitensis*), are smooth-type strains [44,45,46]. It will be much more valuable to investigate the interplay between smooth-type *Brucella* and host cell ferroptosis. Therefore, we attempted to use *Brucella* M5, a smooth-type vaccine strain derived from virulent *B. melitensis* M28, to explore whether smooth-type *Brucella* strains trigger the occurrence of ferroptosis in RAW264.7 mouse macrophages and what roles host cell ferroptosis plays in *Brucella* infection.

## 2. Materials and Methods

### 2.1. Reagents and Antibodies

Ferrostatin-1 (Fer-1, #HY-100579), Erastin (#HY-15763), and 2,4-Diamino-6-hydroxypyrimidine (DAHP, #HY-100954) were purchased from MCE (Shanghai, China). A cell counting kit-8 (#C0038), lipid peroxidation MDA assay kit (#S0131), LDH cytotoxicity assay kit (#C0017), and GSSG/GSH quantification kit (#S0053) were purchased from Beyotime (Shanghai, China). A mouse BH4 ELISA kit (#RD-RX28523) and Mouse 4-HNE ELISA kit (#RD-RX21859) were purchased from Henghuibio (Beijing, China). A GPX4 antibody (#ab125066) and a GCH1 antibody (#ab307507) were purchased from Abcam (Cambridge, UK). A p53 antibody (#2524) was purchased from CST (Danvers, MA, USA). A β-Tubulin antibody (#10068-1-AP), GAPDH antibody (#10494-1-AP), AIFM2/FSP1 antibody (#20886-1-AP), HRP-conjugated goat anti-rabbit IgG (#SA00001-2), and HRP-conjugated goat anti-mouse IgG (#SA00001-1) were purchased from Proteintech (Wuhan, China). A DHODH antibody (#A13295) and SLC7A11/xCT antibody (#A2413) were purchased from ABclonal (Wuhan, China).

### 2.2. Bacterial Strains and Cell Lines

*B. melitensis* strain M5 (*Brucella* M5) was a gift from the Chinese Veterinary Culture Collection Center (Beijing, China) and was cultured in tryptic soy broth (TSB; BD-Pharmingen, Franklin Lakes, NJ, USA) or on tryptic soy agar (TSA; BD-Pharmingen) at 37 °C with 5% CO_2_. RAW264.7 macrophages, a mouse leukemia macrophage line commonly used for studying the virulence of *Brucella,* was purchased from American Type Culture Collection (ATCC; Manassas, VA, USA) and maintained in Dulbecco’s Modified Eagle Medium (DMEM; Gibco, Grand Island, NY, USA) supplemented with 10% fetal bovine serum (FBS; Gibco) at 37 °C in a 5% CO_2_ atmosphere.

### 2.3. Cell Counting Kit-8 Assay

To screen appropriate working concentrations of drugs, a cell counting-kit 8 (CCK8) assay was used to determine the cytotoxicity of drugs to RAW264.7 macrophages. Cells were seeded into 96-well plates (1 × 10^4^ cells per well) and maintained for 24 h. Then, the cells were treated with Fer-1, DAHP, or erastin at different concentrations, respectively, for 24 h, 48 h, and 72 h. After incubation, the CCK8 reagent was added to each well and incubated for 1 h at 37 °C in 5% CO_2_. Ultimately, the optical density (OD) values at 450 nm were measured and the cell viability was calculated.

### 2.4. Cytotoxicity of Drugs to Brucella

*Brucella* M5 was cultured in TSB and grown in the exponential phase (OD_600nm_ ≈ 1.0). The bacterial culture was then inoculated into 5 mL of fresh TSB at a ratio of 1:100 and treated with Fer-1 (at a final concentration of 5 μM), DAHP (at a final concentration of 5 mM), erastin (at a final concentration of 1.25 μM), and vehicle mock, respectively. At 24-, 48-, and 72-h post-incubation, 100 μL of each culture was taken to measure the OD_600nm_ and the percentage of bacterial cell viability was then calculated.

### 2.5. Cell Infection Assay

For infections, RAW264.7 macrophages were seeded into 24-well plates at 2 × 10^5^ cells/well or into 6-well plates at 1 × 10^6^ cells/well and maintained for 24 h prior to infection. Parallelly, *Brucella* M5 was inoculated in TSB and grown in the exponential phase. The bacterial suspension was diluted in DMEM supplemented with 1% FBS and added to each well at a multiplicity of infection (MOI) of 100. The infected cells were then centrifuged at 400× *g* for 5 min at room temperature and incubated for 1 h at 37 °C in a 5% CO_2_ atmosphere. After that, the cells were washed three times with phosphate-buffered saline (PBS; Gibco) and treated with 50 μg/mL of gentamycin (#A100304; Sangon, Shanghai, China) for 1 h to kill the uninternalized bacteria. Subsequently, the cells were washed three times with PBS and incubated with a medium containing 25 μg/mL of gentamycin for the desired time points.

### 2.6. Lactate Dehydrogenase Release Assay

The effect of *Brucella* infection on lactate dehydrogenase (LDH) release of RAW 264.7 macrophages was determined using an LDH cytotoxicity assay kit following the manufacturer’s instructions with some modifications. Cells were seeded into 24-well plates and infected with *Brucella* M5 at a MOI of 100 as described above and cells infected without *Brucella* were parallelly set as negative and maximum LDH release control. At 24-, 48-, and 72-h post-infection (hpi), the cell supernatants were collected and reacted with the kit reagents for 30 min and the absorbance at 490 nm was then measured. The percentage of LDH release was calculated as [(OD of LDH Release − OD of Medium Background)/(OD of Maximum LDH Release − OD of Medium Background)] × 100.

### 2.7. ELISA Assay

RAW264.7 macrophages were seeded into 6-well plates and infected with *Brucella* M5 at a MOI of 100 as described above. At 24-, 48-, and 72-h post-infection, the cells were washed three times with PBS and lysed with lysis buffer (#P0013; Beyotime) on ice for 30 min. Then, the lysates were collected to measure the 4-HNE or BH4 levels using specific ELISA kits according to the manufacturer’s instructions.

### 2.8. Measurement of MDA

RAW264.7 macrophages were seeded into 6-well plates and infected with *Brucella* M5 at a MOI of 100 as described above. At 24-, 48-, and 72-h post-infection, the cells were washed three times with PBS and lysed with lysis buffer on ice for 30 min. Then, the lysates were collected to measure the MDA levels using a specific lipid peroxidation MDA assay kit according to the manufacturer’s instructions.

### 2.9. Western Blot Analysis

RAW264.7 macrophages were seeded into 6-well plates and infected with *Brucella* M5 at a MOI of 100 as described above. At 24-, 48-, and 72-h post-infection, the cells were washed three times with PBS and lysed with lysis buffer on ice for 30 min. Then, the proteins were separated on 12.5% sodium dodecyl sulfate-polyacrylamide gel electrophoresis (SDS-PAGE; #PG113; Epizyme, Shanghai, China) and transferred onto nitrocellulose (NC) membranes (#66485; Pall, Port Washington, NY, USA). The membranes were blocked with 5% skim milk in PBST (PBS supplemented with 0.5% Tween-20) for 1 h at room temperature and then incubated with primary antibodies overnight at 4 °C. Subsequently, the membranes were washed 3 times with PBST and incubated with HRP-conjugated secondary antibodies for 2 h at room temperature. After 3 washes, the membranes were then developed using LumiQ HRP substrate solution (#SB-WB012; ShareBio, Shanghai, China). The primary antibodies were diluted in universal antibody diluent (#WB500D; NCM Biotech, Suzhou, China) at 1:1000 and the HRP-conjugated secondary antibodies were diluted in universal antibody diluent at 1:5000.

### 2.10. Bacterial Intracellular Replication Assay

RAW264.7 macrophages were seeded into 24-well plates and infected with *Brucella* M5 at a MOI of 100. At 1-, 24-, 48-, and 72-h post-infection, the cells were washed three times with PBS and lysed with 0.2% Triton X-100 (#ST1723, Beyotime) for 20 min at 37 °C. Then, the lysates were serially diluted in PBS and plated on TSA. After 3 days of incubation, the bacterial colony-forming units (CFUs) were determined.

### 2.11. Bacterial Egress Assay

To determine the role of host ferroptosis in *Brucella* egress, a 24-period bacterial egress quantification procedure was conducted as reported previously [11,47,48]. RAW264.7 macrophages were seeded into 24-well plates and infected with *Brucella* M5 at a MOI of 100. Them, 24 h prior to the desired time points (24-, 48-, and 72-h post-infection), specific cell wells were washed three times with PBS and replaced with an antibiotic-free medium. After a further 24 h incubation, the supernatants were collected and centrifuged at 200× *g* for 5 min, 200× *g* for 5 min, and 310× *g* for 5 min in sequence at room temperature. Then, the supernatants were serially diluted in PBS and plated on TSA. After 3 days of incubation, the bacterial CFUs were determined.

### 2.12. Statistical Analysis

All experiments were independently repeated at least three times and the data are expressed as the mean ± standard deviation (SD). All data were analyzed with SPSS version 25 (SPSS, Inc., Cary, NC, USA). A Student’s *t*-test was used to analyze differences between two data sets and a one-way analysis of variance (ANOVA) with Tukey’s post-test was used to analyze differences between multiple groups. A *p*-value < 0.05 was considered statistically significant.

## 3. Results

### 3.1. Brucella M5-Induced Ferroptosis of RAW264.7 Macrophages at the Late Stage of Infection

**Figure 1:**
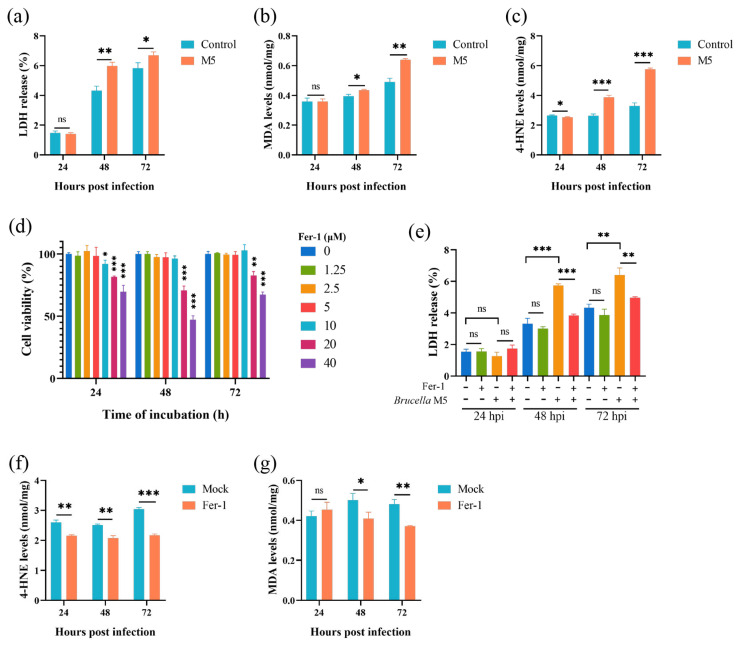
*Brucella* M5 induced ferroptosis of RAW264.7 macrophages at the late stage of infection. (**a**) RAW264.7 macrophages were infected with *Brucella* M5 with a MOI = 100, the LDH release levels were then measured at 24-, 48-, and 72-h post infection; (**b**) RAW264.7 macrophages were infected with *Brucella* M5 with a MOI = 100, the macrophages were then collected and lysed to measure the MDA levels at 24-, 48-, and 72-h post infection; (**c**) RAW264.7 macrophages were infected with *Brucella* M5 with a MOI = 100, the macrophages were then collected and lysed to measure the 4-HNE levels at 24-, 48-, and 72-h post infection; (**d**) RAW264.7 macrophages were treated with Fer-1 at different concentrations for specific times and the cell viability of macrophages were then measured using a CCK8 assay; (**e**) RAW264.7 macrophages were treated with Fer-1 at a final concentration of 5 μM and then infected with *Brucella* M5 (MOI = 100), the LDH release levels were measured at 24-, 48-, and 72-h post infection; (**f**) RAW264.7 macrophages were treated with Fer-1 at a final concentration of 5 μM and then infected with *Brucella* M5 (MOI = 100), the MDA levels were measured at 24-, 48-, and 72-h post infection; (**g**) RAW264.7 macrophages were treated with Fer-1 at a final concentration of 5 μM and then infected with *Brucella* M5 (MOI = 100), the 4-HNE levels were measured at 24-, 48-, and 72-h post infection. ns, no significance; * *p* < 0.05, ** *p* < 0.01, *** *p* < 0.001.

To determine whether smooth-type *Brucella* infection induces host cell ferroptosis, several known hallmarks were measured here. First of all, we conducted an LDH release assay to assess the effect of *Brucella* M5 infection on host cell death. After infection by *Brucella* M5, the LDH release levels of RAW264.7 macrophages were significantly increased at 48- and 72-h post-infection but not at 24 hpi (Figure 1a). We further measured the expressions of MDA and 4-HNE to assess the lipid peroxidation levels. After infection by *Brucella* M5, the MDA levels in RAW264.7 macrophages were significantly increased at 72 hpi (Figure 1b). Spectacularly, the increase in 4-HNE expression in macrophages occurred as early as 24 h post-infection (Figure 1c). These results suggested that *Brucella* M5 may induce host cell ferroptosis at the late stage of infection. We next treated RAW264.7 macrophages with Fer-1, a specific ferroptosis inhibitor, at a final concentration of 5 μM to confirm our assumption (Figure 1d). Under the condition of Fer-1 treatment, the LDH release induced by *Brucella* was significantly suppressed compared with the mock group, indicating an inhibiting role of Fer-1 in *Brucella*-induced host cell death (Figure 1e). Consistently, the treatment of Fer-1 also inhibited the expressions of MDA and 4-HNE in macrophages induced by *Brucella* M5 (Figure 1f,g). These results indicated that smooth-type *Brucella* can induce host cell ferroptosis, which occurred at the late stage of infection.

### 3.2. Effects of Brucella Infection on Ferroptosis Regulation Systems

**Figure 2:**
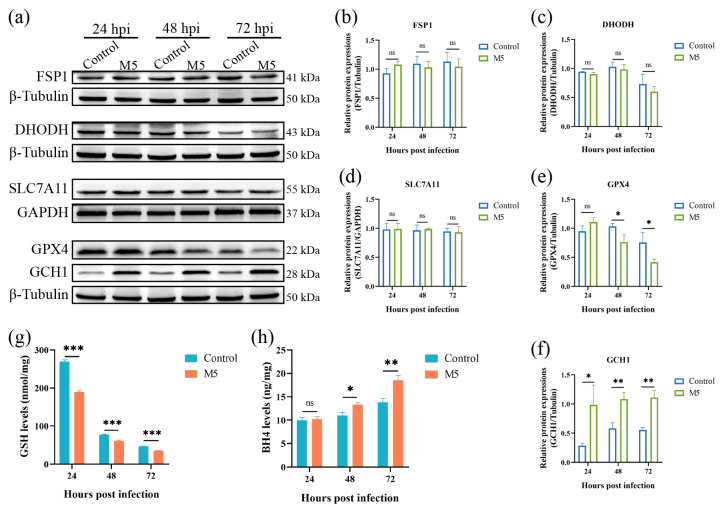
Effect of *Brucella* M5 infection on ferroptosis defense systems in RAW264.7 macrophages. (**a**) RAW264.7 macrophages were infected with *Brucella* M5 with an MOI = 100 for 24, 48, and 72 h; the cells were then collected and lysed to detect the expression of ferroptosis regulation proteins via Western blot; (**b**–**f**) The relative expression levels of target proteins by analyzing the relative intensity of specific protein bands; (**g**) RAW264.7 macrophages were infected with *Brucella* M5 with an MOI = 100, the GSH levels were then measured at 24-, 48-, and 72-h post-infection; (**h**) RAW264.7 macrophages were infected with *Brucella* M5 with an MOI = 100, the BH4 levels were then measured at 24-, 48-, and 72-h post-infection. ns, no significance; * *p* < 0.05, ** *p* < 0.01, *** *p* < 0.001.

Ferroptosis is tightly regulated by multiple regulation pathways. To date, there are four ferroptosis defense pathways that have been characterized; they are the SLC7A11- GPX4-GSH pathway, the FSP1-CoQ10 pathway, the DHODH-CoQ10 pathway, and the GCH1-BH4 pathway. As *Brucella* infection induces the occurrence of host cell ferroptosis, we thus determined the effect of *Brucella* infection on these ferroptosis defenders. The Western blot results showed that the infection of *Brucella* M5 did not affect the expression of FSP1 and DHODH in RAW264.7 macrophages (Figure 2a–c). The expression of SLC7A11 was also insensitive to *Brucella* infection, suggesting that Brucella infection did not influence the import of cystine in RAW264.7 macrophages (Figure 2a,d). However, the expression of GPX4 was significantly suppressed at 48- and 72-h post-infection, which was consistent with the results of the LDH release assay (Figure 2a,e). Strikingly, the expression of GCH1 was significantly up-regulated as early as 24 h post-infection, which was inconsistent with the LDH release assay (Figure 2a,f). Subsequently, we further detected the expressions of GSH and BH4, the executors of the GPX4 and GCH1, respectively. The results showed that the intracellular GSH level was significantly decreased under *Brucella* infection (Figure 2g). Regarding BH4, the BH4 level of RAW264.7 macrophages was increased at 48- and 72-h after *Brucella* infection, which was consistent with the Western blot result that GCH1 was up-regulated by *Brucella* (Figure 2h). These results showed that *Brucella* plays a contradictory role in the GPX4-GSH pathway and the GCH1-BH4 pathway. Cells with high expression of GCH1 are more resistant to ferroptosis [35]. We thus speculate that *Brucella* may increase the GCH1-BH4 pathway to exhibit a ferroptosis-suppression role at the early stage of infection but ultimately induce host cell ferroptosis at the late stage of infection through inhibiting the GPX4-GSH pathway, the predominant defense pathway of ferroptosis.

### 3.3. Inhibiting the GCH1-BH4 Pathway Promoted Brucella-Induced Host Cell Ferroptosis

**Figure 3:**
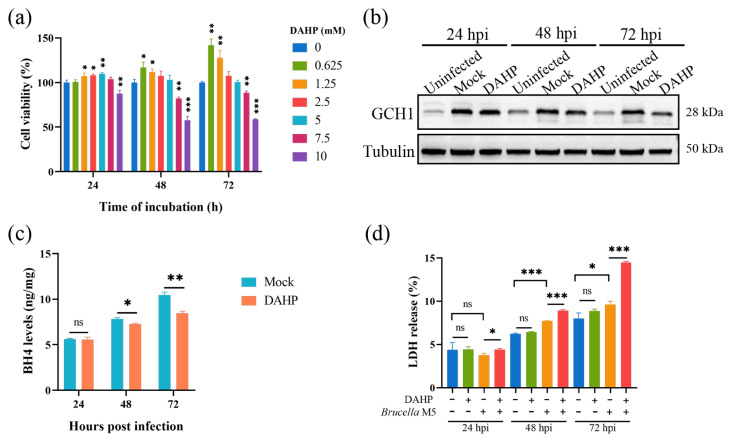
DAHP-mediated GCH1-BH4 axis inhibition up-regulated *Brucella*-induced ferroptosis in RAW264.7 macrophages. (**a**) RAW264.7 macrophages were treated with DAHP at different concentrations for specific times and the cell viability of macrophages was then measured using a CCK8 assay; (**b**) RAW264.7 macrophages were treated with DAHP at a final concentration of 5 mM and then infected with *Brucella* M5 (MOI = 100) for 24, 48, and 72 h, the cells were collected and lysed to detect the protein expression of GCH1 via Western blot; (**c**) RAW264.7 macrophages were treated with DAHP at a final concentration of 5 mM and then infected with *Brucella* M5 (MOI = 100), the BH4 levels were measured at 24-, 48-, and 72-h post-infection; (**d**) RAW264.7 macrophages were treated with DAHP at a final concentration of 5 mM and then infected with *Brucella* M5 (MOI = 100), the LDH release levels were measured at 24-, 48-, and 72-h post-infection. ns, no significance; * *p* < 0.05, ** *p* < 0.01, *** *p* < 0.001.

Here, we attempted to confirm our hypothesis that *Brucella* plays a ferroptosis-inhibiting role at the early stage of infection by increasing the GCH1-BH4 axis. We thus used DAHP, a specific inhibitor for GCH1, to suppress the expression of GCH1 at a concentration of 5 mM (Figure 3a). As shown in Figure 3b, the treatment of 5 mM DAHP decreased the expression of GCH1 at 48- and 72-h post *Brucella* infection. We further determined the effect of DAHP administration on BH4 expression in *Brucella*-infected RAW264.7 macrophages. Consistent with the decrease in GCH1 protein, the BH4 level was also significantly decreased under DAHP treatment (Figure 3c). Subsequently, we assessed the effect of DAHP on *Brucella*-induced cell death of RAW264.7 macrophages. The result of the LDH release assay showed that DAHP administration significantly promoted *Brucella*-induced host cell death at as early as 24 hpi (Figure 3d). These results supported our speculation that *Brucella* inhibits host cell ferroptosis through up-regulating the GCH1-BH4 pathway and that this inhibition effect is mainly exhibited at the early stage of infection.

### 3.4. Inhibiting Host Cell Ferroptosis Promoted Brucella Intracellular Replication

**Figure 4:**
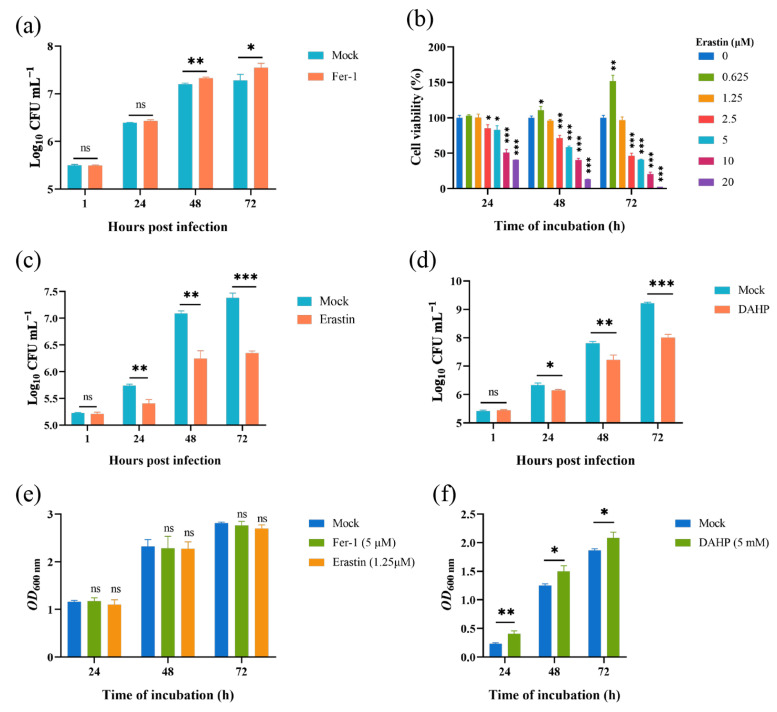
Host cell ferroptosis was unfavorable for *Brucella* intracellular replication. (**a**) RAW264.7 macrophages were treated with Fer-1 at a final concentration of 5 μM and then infected with *Brucella* M5 (MOI = 100), the cells were subsequently lysed to determine the intracellular number of *Brucella* at 1, 24-, 48-, and 72-h post infection; (**b**) RAW264.7 macrophages were treated with erastin at different concentrations for specific times, the cell viability of macrophages were then measured using a CCK8 assay; (**c**) RAW264.7 macrophages were treated with erastin at a final concentration of 1.25 μM and then infected with *Brucella* M5 (MOI = 100), the cells were subsequently lysed to determine the intracellular number of *Brucella* at 1, 24-, 48-, and 72-h post infection; (**d**) RAW264.7 macrophages were treated with DAHP at a final concentration of 5 mM and then infected with *Brucella* M5 (MOI = 100), the cells were subsequently lysed to determine the intracellular number of *Brucella* at 1, 24-, 48-, and 72-h post infection; (**e**,**f**) *Brucella* M5 was treated with Fer-1, erastin, and DAHP at a final concentration of 5 μM, 5 mM, and 1.25 μM, respectively. At 24-, 48-, and 72-h post-treatment, the OD_600nm_ of bacterial culture from each group was measured. ns, no significance; * *p* < 0.05, ** *p* < 0.01, *** *p* < 0.001.

Given that the *Brucella* M5 strain modulated host cell ferroptosis during infection, we wonder whether host cell ferroptotic stress plays a role in *Brucella* intracellular replication. Therefore, we treated RAW264.7 macrophages with Fer-1 and then measured the intracellular replication by plating colony-counting methods. As shown in Figure 4a, the administration of Fer-1 significantly increased the intracellular survival of *Brucella* at 48- and 72-h post-infection, indicating that the suppression of host cell ferroptosis promotes *Brucella* intracellular replication. On the other hand, we assessed the effect of elevating host cell ferroptosis on *Brucella* intracellular survival. We first treated RAW264.7 macrophages with a specific ferroptosis inducer, erastin, at a concentration of 1.25 μM to elevate the ferroptotic stress (Figure 4b). The result showed that the administration of erastin decreased *Brucella* intracellular survival at 24-, 48-, and 72-h post-infection, suggesting an inhibitory role of elevated ferroptosis in *Brucella* intracellular survival (Figure 4c). To confirm the result, we treated RAW264.7 macrophages with DAHP to promote ferroptosis by inhibiting the GCH1-BH4 axis. The result showed that DAHP treatment also decreased the *Brucella* intracellular survival at 24-, 48-, and 72-h post-infection (Figure 4d). Next, we assessed the cytotoxic of Fer-1, DAHP, and erastin for *Brucella* M5 to eliminate the possibility of drugs affecting the cell viability of *Brucella* M5. The result showed that the drugs did not affect the cell viability of *Brucella* M5 at the specific concentrations used in this study (Figure 4e,f). These results indicated that host cell ferroptosis is harmful to *Brucella* intracellular replication.

### 3.5. Inhibiting Host Cell Ferroptosis Hampered Brucella Egress

**Figure 5:**
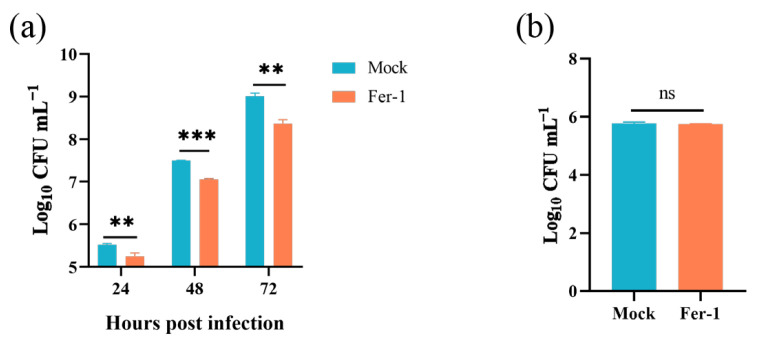
Inhibition of ferroptosis decreased *Brucella* egress from RAW264.7 macrophages. (**a**) RAW264.7 macrophages were treated with Fer-1 at a final concentration of 5 μM and infected with *Brucella* M5 (MOI = 100); 24 h prior to the required time points, cells were washed and incubated with antibiotic-free medium. After further 24 h incubation, the supernatants were collected to determine the *Brucella* numbers; (**b**) RAW264.7 macrophages were treated with Fer-1 at a final concentration of 5 μM and then infected with *Brucella* M5 (MOI = 100), the cells were subsequently lysed to determine the intracellular number of *Brucella* at 1 hpi. ns, no significance; ** *p* < 0.01, *** *p* < 0.001.

In this part, we explored the role of host cell ferroptosis in the egress of intracellular *Brucella*. Elevated ferroptosis inhibits *Brucella* intracellular replication, which will affect the bacteria numbers egressed from host cells. Thus, here, we determined the effect of Fer-1-mediated ferroptosis suppression on *Brucella* egress. As shown in Figure 5a, the administration of 5 μM Fer-1 significantly decreased the egress of intracellular *Brucella* as early as 24-h post-infection. Moreover, similar to the intracellular survival assay mentioned above, the treatment of Fer-1 did not affect the invasion of *Brucella* (Figure 5b). These results indicated that host cell ferroptosis facilitates *Brucella* egress and dissemination.

## 4. Discussion

*Brucella* is a facultative intracellular pathogenic bacteria; its virulence is considered completely dependent on its capability to survive and replicate within host cells [11]. In the in vitro model, the intracellular life cycle of *Brucella* consists of three stages: the arrested stage (about the first 12 h post-infection), the replication stage (about 12~48 h post-infection), and the egress stage (about 48~72 h post-infection) [4,13]. The last stage is the pivotal one for the intracellular life of *Brucella*, which facilitates the pathogen’s dissemination and reinfection. Celli and colleagues initiatively reported that the egress of *Brucella* relies on the formation of an autophagic-like *Brucella*-containing-vacuole (aBCV), a membrane-bound compartment containing autophagy markers such as ULK1 and Beclin1 [49]. Perturbating or blocking the aBCV formation or maturation will decrease the egress of *Brucella* from host cells [48,50,51]. Recent work indicated that, except for the aBCV-dependent egress path, a subpopulation of the intracellular *Brucella* takes advantage of host cells’ multivesicular bodies to egress [11]. This report indicates that *Brucella* may have multiple strategies to egress and spread. Noteworthily, it has been reported that host cell ferroptosis is involved in intracellular pathogen dissemination. Amaral et al. found that *M. tuberculosis* induces host ferroptosis both in and ex vivo [39]. Moreover, the induction of host ferroptosis facilitates the dissemination of *M. tuberculosis* [39,40]. Similarly, our present results showed that *Brucella* M5 induced macrophage ferroptosis at the late stage of infection by suppressing the GPX4-GSH pathway. Furthermore, the pharmacological inhibition of host cell ferroptosis decreased the egress of *Brucella* from macrophages, suggesting that host cell ferroptosis may be an additional route for *Brucella* to egress and spread. This finding provides a novel insight into the pathogenic mechanisms of *Brucella*.

Ferroptosis is a novel form of programmed cell death characterized by the aberrant accumulation of lipid peroxides [22]. Programmed cell death is considered a defensive strategy for the host to restrict certain pathogenic infections by eliminating their intracellular inches [38,52]. Recent studies have reported that host cell ferroptosis is widely involved in constraining bacteria intracellular survival [36,37,38]. *Ehrlichia chaffeensis* (*E. chaffeensis*) is an intracellular pathogen and its intracellular proliferation needs iron. Inside host cells, *E. chaffeensis* robs host iron from ferritin through secreting *Ehrlichia* translocated factor-3 (Etf-3) into a host cell to trigger ferritinophagy. Meanwhile, to prevent ferritinophagy-induced ROS accumulation and subsequent cell damage, *E. chaffeensis* translocates another effector, *Ehrlichia* translocated factor-1 (Etf-1), into host cell mitochondria and reduces cellular ROS by stabilizing the mitochondrial matrix [53]. Ma et al. reported that *S. aureus*, *E. coli,* and *S. pullorum* can trigger host ferroptotic stress at the early stage of infection and this stress promotes the elimination of intracellular bacteria [42]. In the present study, we showed that *Brucella* M5 promotes the GCH1-BH4 axis during infecting macrophages to exhibit a ferroptosis-inhibiting effect at the early infection phase. Our results further indicated that drug treatments that induce ferroptosis inhibited the intracellular replication of *Brucella*, while ferroptosis inhibitor promoted its intracellular replication, which was consistent with previous reports that host cell ferroptosis restricts the intracellular survival of bacterial pathogens. Given that the capability to survive and replicate in host cells is the determinant of *Brucella* virulence [9,11] and numerous studies reported that *Brucella* infection prevents the programmed cell death of monocyte, lymphocyte, macrophages, and lung epithelial cells, even the non-infected neighbor cells are also protected from regulated cell death [16,17,19,20], we raise an assumption that *Brucella* inhibits host cell ferroptosis at the early stage of infection to facilitate its intracellular multiplication. Thus, modulating host cell death, including ferroptosis, may be one of the strategies utilized by *Brucella* to facilitate its intracellular replication.

Ferroptosis is dependent on numerous molecular bases and is tightly regulated by multiple regulation pathways. Although our present study revealed a noticeable role of host cell ferroptosis in *Brucella* intracellular replication and egress and demonstrated the effect of *Brucella* on the GPX4-GSH and GCH1-BH4 pathways, the interplay between *Brucella* infection and host cell ferroptosis still has many more gaps. As is evident from the definition of ferroptosis, iron is essential for the initiation and propagation of lipid peroxidation [23,54]. The peroxidation of membrane-anchored PUFA-containing lipids, the precursor of lipid hydroperoxides, can be catalyzed enzymatically or non-enzymatically, both of which require iron [22,27,54,55,56]. Hop et al. reported that *Brucella* secretes Dps, a DNA-binding protein, into a host cell to trigger ferritinophagy, which elevates the intracellular iron levels to promote *Brucella* growth [57]. However, the iron-catalyzed Fenton reaction is highly toxic for host cells and the subsequent oxidative burst is the most pronounced defense mechanism in *Brucella*-infected cells [58]. Moreover, the supplementation of ferrous iron prevents *Brucella* from egressing [48]. These findings suggest a substantial interplay between *Brucella* infection and host cell iron homeostasis. Dar et al. discovered that *P. aeruginosa* can trigger ferroptosis in human bronchial epithelial cells by expressing a lipoxygenase to oxidize host arachidonic acid-phosphatidylethanolamines [41]. *E. chaffeensis* translocate multiple effectors into host cells to balance ferritinophagy and ROS generation, thereby robbing cellular iron to feed its intracellular growth [53]. *M. tuberculosis* translocated an effector, PtpA, into the host to trigger ferroptosis by inhibiting GPX4 expression, which eventually promotes bacteria dissemination [40]. The *virB* operon encoded type IV secretion system (T4SS), which functions by translocating effectors into host cells, is essential for *Brucella* intracellular survival and contributes to the completion of bacterial egress [4,10,50]. What if *Brucella* modulates host cell ferroptosis using an effector-dependent manner? Collectively, there are many more issues that need to be explored in the future, for example, whether and how *Brucella* modulates the molecular basis of host cell ferroptosis, what is the molecular mechanism for *Brucella* manipulating specific ferroptosis pathways, and whether and how *Brucella*-specific effectors or molecules participate in the interaction between *Brucella* infection and host cell ferroptosis.

In this study, our findings were primarily obtained from *Brucella*-infected RAW264.7 cell line. This cell line originates from murine tumors induced with *Abelson leukemia* virus, which exhibits the properties of macrophages, such as pinocytosis, phagocytosis, and inflammatory and immunomodulatory responses to stimuli [59,60,61]. Compared to primary macrophages, the RAW264.7 cell line has many advantages: wide availability, easy to operate, homogeneous genetic background, and functional stability [62,63,64]. Therefore, the RAW264.7 cell line is extensively used as a macrophage model for in vitro biology studies [65,66]. Except for that, this cell line also gained popularity as an in vitro model for investigating pathogens’ infection, including *Brucella* [62,63,67,68,69]. Further studies demonstrated that the gene expression profiles and/or stimuli-induced specific pathways of RAW264.7 cell line has both shared parts and different parts from that of primary macrophages, primary bone marrow-derived macrophages, or human macrophage-like cell line THP-1 [64,69,70,71,72]. Therefore, it should be aware of the fact that specific molecular mechanisms that underly the interplay between *Brucella* infection and host cell ferroptosis may be variable in different cell environments. In the other hand, the in vivo infection of *Brucella* involves multiple systems and cell populations and their synergistic or cascading actions. Thus, the findings obtained from a single cell line-based in vitro model may not fully recapitulate the complexity of *Brucella* infection in vivo, leading to the urgency of a systemic investigation on the interaction between *Brucella* and the host. Even so, our findings uncover an important role of host ferroptosis in *Brucella* infection. Currently, the treatment of human brucellosis relies on the combinations of antibiotics but these antibiotics have nonnegligible side effects [2]. Given the roles of host ferroptosis in the intracellular life cycle of *Brucella*, drugs or molecules that target to ferroptosis may have the potential to aid brucellosis treatment. In addition, considering the changes in specific ferroptosis pathways, a clinical potential of host ferroptosis in brucellosis diagnosis could be forecasted.

**Figure 6:**
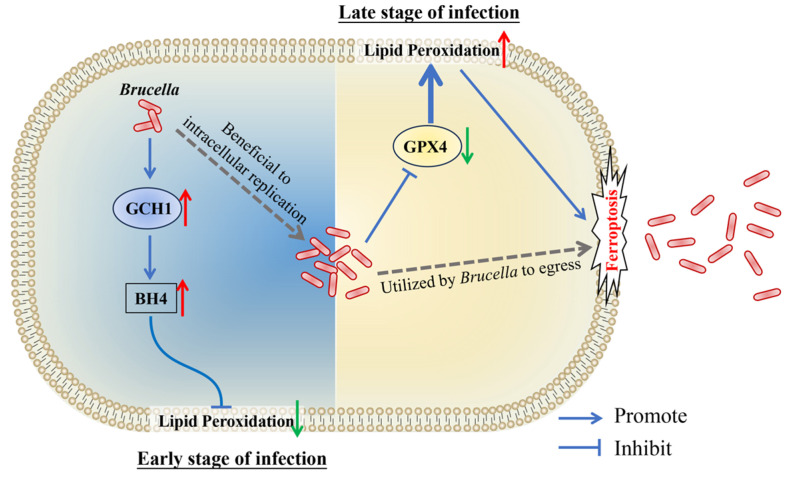
Schematic diagram of *Brucella* manipulates host cell ferroptosis to facilitate its intracellular replication and egress. The red up arrows present up-regulation, the green down arrows present down-regulation.

In conclusion, our present study revealed that *Brucella* M5 suppresses macrophage ferroptosis to benefit its intracellular replication at the early stage of infection through promoting the GCH1-BH4 pathway but induces host cell ferroptosis to promote its egress from macrophages at the late stage of infection by inhibiting the GPX4-GSH axis (Figure 6). These findings provide insights into the role of host cell ferroptosis in *Brucella* infection, which will extend our knowledge about the underlying mechanism of how *Brucella* completes its intracellular life cycle.

## Data Availability

All the data are available within the article.

## References

[1] Comerci D.J., Martinez-Lorenzo M.J., Sieira R., Gorvel J.P., Ugalde R.A. (2001). Essential role of the VirB machinery in the maturation of the *Brucella abortus*-containing vacuole. Cell Microbiol..

[2] Franco M.P., Mulder M., Gilman R.H., Smits H.L. (2007). Human brucellosis. Lancet Infect. Dis..

[3] Dean A.S., Crump L., Greter H., Schelling E., Zinsstag J. (2012). Global Burden of Human Brucellosis: A Systematic Review of Disease Frequency. PLoS Neglected Trop. Dis..

[4] Celli J. (2019). The Intracellular Life Cycle of *Brucella* spp.. Microbiol. Spectr..

[5] Godfroid J., Al Dahouk S., Pappas G., Roth F., Matope G., Muma J., Marcotty T., Pfeiffer D., Skjerve E. (2013). A “One Health” surveillance and control of brucellosis in developing countries: Moving away from improvisation. Comp. Immunol. Microb..

[6] Hull N.C.S., Brant A. (2018). Comparisons of brucellosis between human and veterinary medicine. Infect. Ecol. Epidemiol..

[7] Laine C.G., Johnson V.E., Scott H.M., Arenas-Gamboa A.M. (2023). Global Estimate of Human Brucellosis Incidence. Emerg. Infect. Dis..

[8] Franc K.A., Krecek R.C., Hasler B.N., Arenas-Gamboa A.M. (2018). Brucellosis remains a neglected disease in the developing world: A call for interdisciplinary action. BMC Public Health.

[9] Pei J.W., Turse J.E., Wu Q.M., Ficht T.A. (2006). *Brucella abortus* rough mutants induce macrophage oncosis that requires bacterial protein synthesis and direct interaction with the macrophage. Infect. Immun..

[10] Celli J., de Chastellier C., Franchini D.M., Pizarro-Cerda J., Moreno E., Gorvel A.P. (2003). Brucella evades macrophage killing via VirB-dependent sustained interactions with the endoplasmic reticulum. J. Exp. Med..

[11] Spera J.M., Guaimas F., Czibener C., Ugalde J.E. (2023). Brucella Egresses from Host Cells Exploiting Multivesicular Bodies. MBio.

[12] Zhang G.D., Zhong F.L., Chen L., Qin P.P., Li J.M., Zhi F.J., Tian L.L., Zhou D., Lin P.F., Chen H.T. (2021). Integrated Proteomic and Transcriptomic Analyses Reveal the Roles of Brucella Homolog of BAX Inhibitor 1 in Cell Division and Membrane Homeostasis of Brucella suis S2. Front. Microbiol..

[13] Marchesini M.I., Spera J.M., Comerci D.J. (2024). The ‘ins and outs’ of Brucella intracellular journey. Curr. Opin. Microbiol..

[14] Barquero-Calvo E., Mora-Cartin R., Arce-Gorvel V., de Diego J.L., Chacon-Diaz C., Chaves-Olarte E., Guzman-Verri C., Buret A.G., Gorvel J.P., Moreno E. (2015). *Brucella abortus* Induces the Premature Death of Human Neutrophils through the Action of Its Lipopolysaccharide. PLoS Pathog..

[15] Gutierrez-Jimenez C., Mora-Cartin R., Altamirano-Silva P., Chacon-Diaz C., Chaves-Olarte E., Moreno E., Barquero-Calvo E. (2019). Neutrophils as Trojan Horse Vehicles for *Brucella abortus* Macrophage Infection. Front. Immunol..

[16] Gross A., Terraza A., Ouahrani-Bettache S., Liautard J.P., Dornand J. (2000). In vitro Brucella suis infection prevents the programmed cell death of human monocytic cells. Infect. Immun..

[17] Tolomeo M., Di Carlo P., Abbadessa V., Titone L., Miceli S., Barbusca E., Cannizzo G., Mancuso S., Arista S., Scarlata F. (2003). Monocyte and lymphocyte apoptosis resistance in acute and chronic brucellosis and its possible implications in clinical management. Clin. Infect. Dis..

[18] He Y.Q., Reichow S., Ramamoorthy S., Ding X.C., Lathigra R., Craig J.C., Sobral B.W.S., Schurig G.G., Sriranganathan N., Boyle S.M. (2006). Brucella melitensis triggers time-dependent modulation of a apoptosis and down-regulation of mitochondrion-associated gene expression in mouse macrophages. Infect. Immun..

[19] Barquero-Calvo E., Chaves-Olarte E., Weiss D.S., Guzman-Verri C., Chacon-Diaz C., Rucavado A., Moriyon I., Moreno E. (2007). *Brucella abortus* uses a stealthy strategy to avoid activation of the innate immune system during the onset of infection. PLoS ONE.

[20] Ferrero M.C., Fossati C.A., Baldi P.C. (2009). Smooth Brucella strains invade and replicate in human lung epithelial cells without inducing cell death. Microbes Infect..

[21] Cui G.M., Wei P., Zhao Y.X., Guan Z.H., Yang L., Sun W.C., Wang S.X., Peng Q.S. (2014). Brucella infection inhibits macrophages apoptosis via Nedd4-dependent degradation of calpain2. Vet. Microbiol..

[22] Dixon S.J., Lemberg K.M., Lamprecht M.R., Skouta R., Zaitsev E.M., Gleason C.E., Patel D.N., Bauer A.J., Cantley A.M., Yang W.S. (2012). Ferroptosis: An Iron-Dependent Form of Nonapoptotic Cell Death. Cell.

[23] Hassannia B., Van Coillie S., Vanden Berghe T. (2021). Ferroptosis: Biological Rust of Lipid Membranes. Antioxid. Redox Signal..

[24] Wahida A., Conrad M. (2023). Ferroptosis: Under pressure!. Curr. Biol..

[25] Conrad M., Pratt D.A. (2019). The chemical basis of ferroptosis. Nat. Chem. Biol..

[26] Yan H.F., Zou T., Tuo Q.Z., Xu S., Li H., Belaidi A.A., Lei P. (2021). Ferroptosis: Mechanisms and links with diseases. Signal Transduct. Target. Ther..

[27] Wang B., Wang Y., Zhang J., Hu C., Jiang J., Li Y., Peng Z. (2023). ROS-induced lipid peroxidation modulates cell death outcome: Mechanisms behind apoptosis, autophagy, and ferroptosis. Arch. Toxicol..

[28] Yang W.S., SriRamaratnam R., Welsch M.E., Shimada K., Skouta R., Viswanathan V.S., Cheah J.H., Clemons P.A., Shamji A.F., Clish C.B. (2014). Regulation of ferroptotic cancer cell death by GPX4. Cell.

[29] Naowarojna N., Wu T.W., Pan Z.J., Li M.Y., Han J.R., Zou Y.L. (2023). Dynamic Regulation of Ferroptosis by Lipid Metabolism. Antioxid. Redox Signal..

[30] Bersuker K., Hendricks J.M., Li Z., Magtanong L., Ford B., Tang P.H., Roberts M.A., Tong B., Maimone T.J., Zoncu R. (2019). The CoQ oxidoreductase FSP1 acts parallel to GPX4 to inhibit ferroptosis. Nature.

[31] Doll S., Freitas F.P., Shah R., Aldrovandi M., da Silva M.C., Ingold I., Grocin A.G., da Silva T.N.X., Panzilius E., Scheel C.H. (2019). FSP1 is a glutathione-independent ferroptosis suppressor. Nature.

[32] Mao C., Liu X.G., Zhang Y.L., Lei G., Yan Y.L., Lee H., Koppula P., Wu S.Q., Zhuang L., Fang B.L. (2021). DHODH-mediated ferroptosis defence is a targetable vulnerability in cancer. Nature.

[33] Kraft V.A.N., Bezjian C.T., Pfeiffer S., Ringelstetter L., Muller C., Zandkarimi F., Merl-Pham J., Bao X., Anastasov N., Kossl J. (2020). GTP Cyclohydrolase 1/Tetrahydrobiopterin Counteract Ferroptosis through Lipid Remodeling. ACS Cent. Sci..

[34] Soula M., Weber R.A., Zilka O., Alwaseem H., La K., Yen F., Molina H., Garcia-Bermudez J., Pratt D.A., Birsoy K. (2020). Metabolic determinants of cancer cell sensitivity to canonical ferroptosis inducers. Nat. Chem. Biol..

[35] Stockwell B.R. (2022). Ferroptosis turns 10: Emerging mechanisms, physiological functions, and therapeutic applications. Cell.

[36] Amaral E.P., Namasivayam S. (2021). Emerging Role for Ferroptosis in Infectious Diseases. Adv. Exp. Med. Biol..

[37] Bagayoko S., Meunier E. (2021). Emerging roles of ferroptosis in infectious diseases. FEBS J..

[38] Gao J., Wang Q.B., Tang Y.D., Zhai J.B., Hu W., Zheng C.F. (2023). When ferroptosis meets pathogenic infections. Trends Microbiol..

[39] Amaral E.P., Costa D.L., Namasivayam S., Riteau N., Kamenyeva O., Mittereder L., Mayer-Barber K.D., Andrade B.B., Sher A. (2019). A major role for ferroptosis in Mycobacterium tuberculosis-induced cell death and tissue necrosis. J. Exp. Med..

[40] Qiang L., Zhang Y., Lei Z., Lu Z., Tan S., Ge P., Chai Q., Zhao M., Zhang X., Li B. (2023). A mycobacterial effector promotes ferroptosis-dependent pathogenicity and dissemination. Nat. Commun..

[41] Dar H.H., Tyurina Y.Y., Mikulska-Ruminska K., Shrivastava I., Ting H.C., Tyurin V.A., Krieger J., St Croix C.M., Watkins S., Bayir E. (2018). Pseudomonas aeruginosa utilizes host polyunsaturated phosphatidylethanolamines to trigger theft-ferroptosis in bronchial epithelium. J. Clin. Investig..

[42] Ma R., Fang L., Chen L., Wang X., Jiang J., Gao L. (2022). Ferroptotic stress promotes macrophages against intracellular bacteria. Theranostics.

[43] Hu H., Zhang G., Tian M., Guan X., Yin Y., Ding C., Yu S. (2023). *Brucella abortus* Rough-Type Mutant Induces Ferroptosis and More Oxidative Stress in Infected Macrophages. Pathogens.

[44] Turse J.E., Pei J.W., Ficht T.A. (2011). Lipopolysaccharide-deficient Brucella variants arise spontaneously during infection. Front. Microbiol..

[45] Zhang M., Han X.G., Liu H.W., Tian M.X., Ding C., Song J., Sun X.Q., Liu Z.P., Yu S.Q. (2013). Inactivation of the ABC transporter ATPase gene in *Brucella abortus* strain 2308 attenuated the virulence of the bacteria. Vet. Microbiol..

[46] Tian M.X., Qu J., Han X.G., Ding C., Wang S.H., Peng D.X., Yu S.Q. (2014). Mechanism of Asp24 Upregulation in *Brucella abortus* Rough Mutant with a Disrupted O-Antigen Export System and Effect of Asp24 in Bacterial Intracellular Survival. Infect. Immun..

[47] Miao Y.X., Li G.J., Zhang X.L., Xu H.X., Abraham S.N. (2015). A TRP Channel Senses Lysosome Neutralization by Pathogens to Trigger Their Expulsion. Cell.

[48] Verbeke J., Fayt Y., Martin L., Yilmaz O., Sedzicki J., Reboul A., Jadot M., Renard P., Dehio C., Renard H.F. (2023). Host cell egress of *Brucella abortus* requires BNIP3L-mediated mitophagy. EMBO J..

[49] Starr T., Child R., Wehrly T.D., Hansen B., Hwang S., Lopez-Otin C., Virgin H.W., Celli J. (2012). Selective Subversion of Autophagy Complexes Facilitates Completion of the Brucella Intracellular Cycle. Cell Host Microbe.

[50] Smith E.P., Miller C.N., Child R., Cundiff J.A., Celli J. (2016). Postreplication Roles of the Brucella VirB Type IV Secretion System Uncovered via Conditional Expression of the VirB11 ATPase. MBio.

[51] Luizet J.B., Raymond J., Lacerda T.L.S., Barbieux E., Kambarev S., Bonici M., Lembo F., Willemart K., Borg J.P., Celli J. (2021). The Brucella effector BspL targets the ER-associated degradation (ERAD) pathway and delays bacterial egress from infected cells. Proc. Natl. Acad. Sci. USA.

[52] Jorgensen I., Rayamajhi M., Miao E.A. (2017). Programmed cell death as a defence against infection. Nat. Rev. Immunol..

[53] Yan Q., Zhang W.Q., Lin M.Q., Teymournejad O., Budachetri K., Lakritz J., Rikihisa Y. (2021). Iron robbery by intracellular pathogen via bacterial effector-induced ferritinophagy. Proc. Natl. Acad. Sci. USA.

[54] Jiang X., Stockwell B.R., Conrad M. (2021). Ferroptosis: Mechanisms, biology and role in disease. Nat. Rev. Mol. Cell Biol..

[55] Wu H., Wang F., Ta N., Zhang T., Gao W. (2021). The Multifaceted Regulation of Mitochondria in Ferroptosis. Life.

[56] Dixon S.J., Pratt D.A. (2023). Ferroptosis: A flexible constellation of related biochemical mechanisms. Mol. Cell..

[57] Hop H.T., Huy T.X.N., Lee H.J., Kim S. (2023). Intracellular growth of Brucella is mediated by Dps-dependent activation of ferritinophagy. EMBO Rep..

[58] Jacob J., Finke A., Mielke M. (2020). Survival of *Brucella abortus* S19 and other Brucella spp. in the presence of oxidative stress and within macrophages. Folia Microbiol..

[59] Raschke W.C., Baird S., Ralph P., Nakoinz I. (1978). Functional macrophage cell lines transformed by Abelson leukemia virus. Cell.

[60] Kong L., Smith W., Hao D. (2019). Overview of RAW264.7 for osteoclastogensis study: Phenotype and stimuli. J. Cell Mol. Med..

[61] Facchin B.M., Dos Reis G.O., Vieira G.N., Mohr E.T.B., da Rosa J.S., Kretzer I.F., Demarchi I.G., Dalmarco E.M. (2022). Inflammatory biomarkers on an LPS-induced RAW 264.7 cell model: A systematic review and meta-analysis. Inflamm. Res..

[62] Monack D.M., Raupach B., Hromockyj A.E., Falkow S. (1996). Salmonella typhimurium invasion induces apoptosis in infected macrophages. Proc. Natl. Acad. Sci. USA.

[63] Huttunen K., Ruotsalainen M., Iivanainen E., Torkko P., Katila M., Hirvonen M. (2000). Inflammatory responses in RAW264.7 macrophages caused by mycobacteria isolated from moldy houses. Environ. Toxicol. Pharmacol..

[64] Li P., Hao Z., Wu J., Ma C., Xu Y., Li J., Lan R., Zhu B., Ren P., Fan D. (2021). Comparative Proteomic Analysis of Polarized Human THP-1 and Mouse RAW264.7 Macrophages. Front. Immunol..

[65] Hartley J.W., Evans L.H., Green K.Y., Naghashfar Z., Macias A.R., Zerfas P.M., Ward J.M. (2008). Expression of infectious murine leukemia viruses by RAW264.7 cells, a potential complication for studies with a widely used mouse macrophage cell line. Retrovirology.

[66] Lucy T.T., Mamun-Or-Rashid A.N.M., Yagi M., Yonei Y. (2022). Serial Passaging of RAW 264.7 Cells Modulates Intracellular AGE Formation and Downregulates RANKL-Induced In Vitro Osteoclastogenesis. Int. J. Mol. Sci..

[67] Barthel R., Feng J., Piedrahita J.A., McMurray D.N., Templeton J.W., Adams L.G. (2001). Stable transfection of the bovine NRAMP1 gene into murine RAW264.7 cells: Effect on *Brucella abortus* survival. Infect. Immun..

[68] Eskra L., Mathison A., Splitter G. (2003). Microarray analysis of mRNA levels from RAW264.7 macrophages infected with *Brucella abortus*. Infect. Immun..

[69] Levenson E.A., Martens C., Kanakabandi K., Turner C.V., Virtaneva K., Paneru M., Ricklefs S., Sosnovtsev S.V., Johnson J.A., Porcella S.F. (2018). Comparative Transcriptomic Response of Primary and Immortalized Macrophages to Murine Norovirus Infection. J. Immunol..

[70] Maurya M.R., Gupta S., Li X., Fahy E., Dinasarapu A.R., Sud M., Brown H.A., Glass C.K., Murphy R.C., Russell D.W. (2013). Analysis of inflammatory and lipid metabolic networks across RAW264.7 and thioglycolate-elicited macrophages. J. Lipid Res..

[71] Heffron S.P., Weinstock A., Scolaro B., Chen S., Sansbury B.E., Marecki G., Rolling C.C., El Bannoudi H., Barrett T., Canary J.W. (2021). Platelet-conditioned media induces an anti-inflammatory macrophage phenotype through EP4. J. Thromb. Haemost..

[72] Qie J., Liu Y., Wang Y., Zhang F., Qin Z., Tian S., Liu M., Li K., Shi W., Song L. (2022). Integrated proteomic and transcriptomic landscape of macrophages in mouse tissues. Nat. Commun..

